# Food Protein-Induced Enterocolitis Syndrome Across Lifespan: Focus on Adolescence

**DOI:** 10.3390/jcm14165799

**Published:** 2025-08-16

**Authors:** Marta Barbato, Mariannita Gelsomino, Giulia Bersani, Francesco Mastellone, Valentina Giorgio, Ludovica Iezzi, Rosa Buonagura, Cristiano Caruso, Stefano Miceli Sopo, Angela Rizzi

**Affiliations:** 1 Pediatric Allergy Unit, Department of Life Sciences and Public Health, University Foundation Policlinico Gemelli IRCCS, Catholic University of the Sacred Heart, 00168 Rome, Italy; marta.barbato@guest.policlinicogemelli.it (M.B.); mariannita.gelsomino@gmail.com (M.G.); giuliabersani83@hotmail.com (G.B.); valentinagiorgio1@gmail.com (V.G.); ludovica_iezzi@yahoo.it (L.I.); stefano.micelisopo@unicatt.it (S.M.S.); 2 Department of Internal Medicine and Clinical Complexity, Division of Internal Medicine and Clinical Immunology, Azienda Ospedaliera Universitaria Federico II, 80131 Naples, Italy; buonagura.rosa@gmail.com; 3 UOSD Allergologia e Immunologia Clinica, Dipartimento Scienze Mediche e Chirurgiche, Fondazione Policlinico Universitario A. Gemelli IRCCS, 00168 Rome, Italy; cristiano.caruso@policlinicogemelli.it

**Keywords:** food protein-induced enterocolitis syndrome, food allergy, non-IgE-mediated food allergy, children, adolescents, young adults, diagnostic criteria, biomarkers, transition medicine

## Abstract

**Background/Objectives:** Food protein-induced enterocolitis syndrome (FPIES) is a food-related hypersensitivity disorder characterized by delayed repeated vomiting that typically presents within the first years of life. Although FPIES has traditionally been considered a pediatric condition, it has more recently been observed also in teenagers and adults. Adult FPIES may be a continuation of childhood-onset disease or new-onset forms developing later in life. This review aims to describe the peculiarities of FPIES across the lifespan and to provide an update from the last years on the studies focused on FPIES in adolescence. **Methods:** Papers focusing on FPIES in adolescents, in English and published in PubMed, were reviewed. **Results:** There is less data available in the literature on FPIES in adolescents. Multiple sensitizations to food can compromise nutritional status in patients with FPIES. Several potential diagnostic biomarkers related to genomic susceptibility, altered immunologic response, mucosal inflammation and intestinal microbiota are under study/validation. The lack of age-specific diagnostic algorithms makes it difficult to understand the clinical features of persistent forms of FPIES. **Conclusions:** Shared transition medicine protocols tailored to adolescents could help us better understand the clinical, pathophysiological, diagnostic, and therapeutic characteristics of this delicate phase of life.

## 1. Introduction

Food protein-induced enterocolitis syndrome (FPIES) has traditionally been considered a food allergy that mainly affects infants and young children. However, increasing evidence supports the identification of this condition even in adolescents and adults [1].

The pediatric FPIES is a non-IgE-mediated food allergy, and its onset is typically in the first years of life [2,3].

In pediatric FPIES, episodes of repeated vomiting occur 1 to 4 h after ingestion of the offending food, sometimes accompanied by pallor, hypotonia, and lethargy, and occasionally followed by diarrhea [4]. However, vomiting is not pathognomonic and may also occur in other conditions, such as viral gastroenteritis, sepsis, eosinophilic gastrointestinal disorders, and even metabolic diseases. The diagnosis is clinical, and several panels of diagnostic criteria have been proposed over time [4,5,6,7].

In adults, the most common symptom is abdominal pain, followed by diarrhea. In contrast, vomiting, typically considered the main diagnostic criterion of pediatric FPIES, is absent in one-quarter of the adult population [1]. All three symptoms are observed in only about 30% of adult patients [8].

Little data is available in the literature on FPIES in adolescents, in which this food allergy can present in two different clinical forms. The first form, called “persistent FPIES”, developed during childhood and continued into later years [8]. There is no exhaustive data in the literature regarding the clinical features of these forms [9]. The other form of FPIES, “adolescent new onset FPIES”, can occur for the first time in teenagers and young adults, with clinical manifestations like those observed in adults [9].

Diagnosing acute FPIES in pediatric patients can be complex, as its clinical features are often nonspecific and may resemble conditions such as viral gastroenteritis, celiac disease, or IgE-mediated food allergies. While vomiting is a hallmark symptom, it is not exclusive to FPIES, highlighting the importance of comprehensive history-taking and accurate differential diagnosis.

The 2017 international criteria define acute FPIES by repetitive vomiting 1–4 h after food ingestion without IgE-mediated signs, plus at least three minor symptoms (e.g., pallor, lethargy, diarrhoea) [4]. Newer criteria introduced in 2021 and 2024 include scoring systems and expanded definitions to help identify milder or atypical cases [5,7].

When the diagnosis is uncertain, especially after a single episode, oral food challenge (OFC) remains the gold standard. Protocols vary: the 2017 guidelines suggest 0.06–0.6 g of protein in three doses over 30 min, with 4–6 h of monitoring [4], while others use variations based on age-appropriate portions [10].

Atypical FPIES, marked by delayed vomiting and positive IgE tests (e.g., Skin Prick Tests), is more persistent and can occur in older children or adults, particularly with fish and shellfish [11]. Rarely, FPIES may evolve into IgE-mediated allergy or vice versa [12].

Before OFC, testing for specific IgE is advised. When commercial extracts are inadequate, Prick-by-Prick testing with fresh food may be useful, especially for labile proteins [13].

Tolerance in FPIES varies by trigger food and region. Cow’s milk and soy often resolve by age 2 years in countries like Japan, Israel, and Korea [4,14,15,16], but later in the U.S. [4]. Egg and fish-induced FPIES tend to persist longer, with wide geographic variation. In Italy, average ages of tolerance are 24 months for milk, 30–44 months for egg, and 53 months for fish [17,18,19].

In adults, FPIES typically begins around age 35 and shows a strong female predominance (80%) [8]. Common triggers are shellfish (60%), particularly bivalve mollusks, and fish (48%) [20,21]. Unlike in children, vomiting is less common; most adults present with abdominal pain (100%) and diarrhoea (72%) [1], along with symptoms like weakness, chills, and lethargy. Hypothermia, hypotension, and dehydration are rarer [7]. Symptoms can appear up to 6 h after ingestion [7].

Current diagnostic criteria may not suit adults, making oral food challenge (OFC) essential [9]. OFCs often follow pediatric protocols, though dosing varies; some use full adult portions on non-consecutive days [22]. Many adults decline OFC due to prior severe reactions.

Tolerance develops in under 50% of adults within 1–4 years [20,23]; strict food avoidance remains the main treatment. An atopic background (e.g., allergic rhinitis, asthma, eczema) is common in both adult and pediatric patients [9,24].

FPIES can also onset during adolescence (10–19 years), often as a continuation of childhood disease, though it can begin de novo with either pediatric- or adult-like symptoms [25,26]. Persistence beyond childhood is linked to multiple food sensitizations and poor nutritional status from trigger foods like milk and banana [27,28,29].

Adolescent onset may be triggered by new food exposures (e.g., fish, shellfish) [25,30]. Biomarkers under study include genetic variants (DGKZ, SIRPA, RBM8A, ATG16L1), fecal EDN, calprotectin, f-sIgA, cytokines (IL-17, IL-10, CXCL10), and microbiota alterations [31,32,33,34,35,36,37,38,39,40,41,42,43,44,45]. IgA responses vary between FPIES and IgE-mediated allergies, with potential protective roles for f-sIgA [33,35,38].

Treatment remains strict food avoidance [46]. OFC remains the gold standard for diagnosis and assessing tolerance but should be performed in controlled settings [47]. Age-specific diagnostic tools are needed [48,49].

Emerging therapies include stepwise reintroduction protocols, such as the Canadian Egg Ladder, which showed good outcomes in mild cases: 90.5% of children tolerated cooked egg after a median of 7 months [50]. Experimental oral desensitization has been proposed for persistent cases, though only one successful case has been reported [46]. For adolescent and adult patients, however, strict avoidance remains the mainstay of treatment, as desensitization has not been studied in these age groups.

Adolescence is a period of life that generally begins around 10 years of age and continues until the early 19s, characterized by significant physical, social, emotional, and cognitive changes [51]. These changes affect disease perception, adherence to care, and interaction with healthcare systems. Adolescents with chronic conditions such as food allergies may experience increased psychological burden, identity conflict, and risk-taking behavior, including treatment refusal or dietary non-compliance [52,53,54,55,56,57].

Adolescence begins with sexual maturity, or the onset of puberty, which is, in turn, induced by biological factors (e.g., changes in height and growth, increased strength and endurance, and development of sexual attributes). These changes can have both positive and negative psychological effects (e.g., self-doubt, shame, vulnerability, withdrawal, desire for independence, etc.). Furthermore, adolescents are viewed as such by adults and no longer as children (a change in the perception of others) [58]. Different behavior is expected of them. Generally, adolescence ends when the adolescent fully assumes his or her social role (at work, in the family and in relationships, as a consumer, a political citizen, etc.) and achieves a relatively independent life situation [52].

Chronic illnesses such as food allergies can interfere with these very goals of adolescents. This can result in symptoms of depression, a tendency toward isolation (school absenteeism, lack of social opportunities), refusal of treatment, a difficult relationship with health services, and risky behaviors (smoking, alcohol, substance use) resulting from the need to assert one’s “normalcy” and independence [53,54,55,56,57].

Recent evidence of a progressive epidemiological increase in food allergies in adolescents (4–7% in the last decade [59,60]) calls on the scientific community to identify and optimize integrated healthcare management solutions for transition.

The term “*transition*” in medicine refers to a continuum of care for young people with chronic conditions, from pediatric to adult health care services [56]. However, since it is not a simple transfer but an expected event that should encourage the autonomy of recovered individuals and identify specific specialists, it must be prepared in advance, at the right time, and with validated and effective organizational and communication methods [56].

Some studies have highlighted that an inadequate transition process for adolescents with food allergies is associated with a deterioration in their health and an impact on quality of life [61,62].

The aim of this narrative review was to describe the peculiarities of FPIES according to age of onset of symptoms (infancy/childhood, adolescence, and adulthood) and to provide an update from the last years on the studies focused on FPIES in adolescence.

## 2. Methods

In this narrative review, we conducted a literature search for papers on FPIES at different ages (pediatric, adolescent, and adult) published from January 2012 (year of the first reported case of FPIES in adults [63]) to May 2025 through the international PubMed library. The following advanced research strategy was used: (“food protein-induced enterocolitis syndrome”) OR (“FPIES”) OR (“non-IgE food allergy”) AND (“neonates”) OR (“children”) OR (“infants”) OR (“childhood”) OR (“adolescents”) OR (“adolescence”) OR (“older children”) OR (“young adults”) OR (“adults”). We selected clinical trials, case reports, clinical studies, supported research, technical reports, and validation studies. Only English papers were included. The exclusion criteria of research strategy were (i) information from other sources such as review, meta-analysis, book chapters, editorials, commentaries, theses, clinical conference reports, and anthologies from research institutes; (ii) studies not subjected to peer review; (iii) veterinary studies or mathematical models; (iv) studies with only the abstract in English but the full text in a different language; and (v) case reports or letters without clinical data. The duplicates were manually and individually verified by two authors (AR and RB). Additional documents were retrieved through manual searches of the references contained in screened papers.

## 3. Results and Discussion

**Figure 1:**
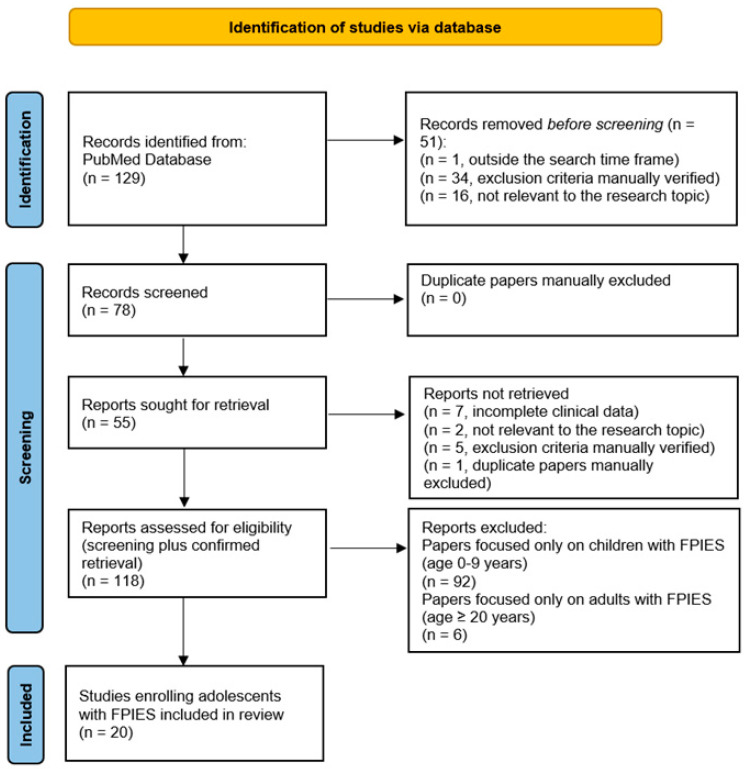
Flowchart of research strategy.

The flowchart of the strategy research is shown in Figure 1. The initial search yielded 129 records from PubMed. After the removal of 51 records for exclusion criteria, not relevance, or time frame, 78 articles were screened. The retrieval process added 40 records. Therefore, 118 records were considered eligible. The final research focused on studies enrolling adolescents with FPIES, which led to the inclusion of 20 records in the review.

### 3.1. FPIES in Infancy/Childhood

In children, a slight male predominance or an equal sex distribution (male 47.5%, female 52.5% [47]) has been observed [8]. The most common trigger food of FPIES in childhood is cow’s milk, followed by fish, egg, rice, soy, corn, poultry, and goat’s milk [17,64]. Regarding the prevalence of culprit foods, there is a difference depending on the geographical area considered. For example, in a retrospective study performed in two Italian hospitals [47], the main foods observed were cow’s milk (30.1%), hen’s egg (21.6%), fish (20.3%), rice (5.5%), wheat (4.7%), and legumes (3.8%), while an Australian population-based study showed that rice (45%) was the most common causative food, followed by cow’s milk (33%), egg (12%), oats (9%), and chicken (8%) [65]. In the Mediterranean population, especially in Italy and Spain, FPIES triggered by a single causative food appears to be predominant [66]. While cow’s milk is the main trigger food in Italy, fish is the predominant single causative food in Spain [19,66]. In fact, in a retrospective study performed in Italy on 66 children with FPIES, 85% of the patients reacted to a single food, while only 15% had multiple reactions [17]. In contrast, in the United States, FPIES to multiple foods, especially grains and legumes, can affect 60–70% of cases [66].

Furthermore, the median age of onset varies depending on the culprit food. The mean age at diagnosis in an Italian retrospective study was 13.2 months for cow’s milk, 17.6 months for hen’s egg, 43.7 months for fish, 10.4 months for rice, and 13.5 months for both wheat and legumes [47].

Over time, several diagnostic criteria panels have been proposed for the diagnosis of acute FPIES, and the issue continues to be debated. However, vomiting is considered the major criterion that must be present to be able to diagnose acute FPIES [4,6].

In fact, according to the 2017 Consensus criteria [4], the major criterion (vomiting in the 1 to 4 h period after ingestion of the suspect food) and at least three of the nine minor criteria (such as pallor, lethargy, or need for emergency department) must be present to meet the diagnosis of acute FPIES. Moreover, there must not be classic IgE-mediated allergic skin or respiratory symptoms. In 2021, Vazquez-Ortiz et al. [5] proposed new diagnostic criteria with a scoring system in which delayed vomiting after ingestion of the suspected food is confirmed as the major criterion, and a second episode of repeated vomiting with the same food makes the diagnosis of acute FPIES much more likely. In addition, new diagnostic and suspicion criteria were proposed in 2024 [7] to make the diagnosis of FPIES even in mild cases that can often escape the criteria of the 2017 Consensus [4].

If only a single episode has occurred, a diagnostic oral food challenge (OFC) should be performed to confirm the diagnosis. In fact, in uncertain cases, OFC remains the gold standard for diagnosis. However, the optimal OFC procedure remains unclear. In fact, there is great heterogeneity in OFC procedures, particularly in cumulative dose, number, size, and timing between doses. The 2017 FPIES Consensus [4] recommends an OFC protocol involving a cumulative protein dose of 0.06 to 0.6 g, usually given in three equal doses over 30 min. The maximum dose is 3 g protein or 10 g total food (or 100 mL liquid), followed by 4 to 6 h of observation. The dosing schedule in children can differ widely: one approach may be to administer 25% of the age-appropriate portion (AAP), followed by 75% of AAP after 4 h [10]. It was also proposed to administer the entire age-appropriate cumulative dose in a single dose with a subsequent 4-h observation [67].

Atypical forms of FPIES have been described in literature. Usually, these patients present symptoms of repeated vomiting delayed after ingestion of the offending food, but Skin Prick Tests (SPTs) are positive and specific serum IgE is detected. However, there are patients who present skin symptoms characteristic of IgE-mediated forms of allergy. In fact, a case was reported describing a patient [68] who presented both clinical expressions of IgE-mediated food allergy and features of FPIES, such as the simultaneous presence of urticaria and an onset of vomiting 2–3 h later, following the ingestion of the culprit food. The clinical course of atypical FPIES in infants may be more persistent or enduring [20]. Notably, atypical FPIES has been described in older children and adults, typically in association with specific foods, such as fish and shellfish [11]. In some cases, FPIES may develop into an IgE-mediated allergy with classic IgE-mediated clinical expression and positive SPT. In fact, an Italian study reported two cases of atypical FPIES evolving into IgE-mediated gastrointestinal anaphylaxis [12]. However, no cases of switch towards IgE-mediated forms have been reported in adults. Conversely, a rare clinical course involving a shift from IgE-mediated cow’s milk allergy to non-IgE-mediated FPIES has been described in a 4-month-old male infant [69]. This progression highlights the necessity of testing specific IgE to the offending food before conducting an oral food challenge in FPIES [69]. Prick-by-Prick (PbP) testing may be used as an alternative to conventional SPT when standardized allergen extracts are unavailable and to diagnose allergies to foods where labile proteins may degrade in commercial extracts [13].

The natural history of FPIES differs according to causal food and region [70]. The age of achieving tolerance in cow’s milk and soy-induced FPIES is earlier than that of solid foods-induced FPIES. Between 60% and 90% of patients with FPIES induced by cow’s milk are tolerant by the age of 2 years in studies conducted in Japan [14], Israel [15], and Australia [4]. In contrast, the tolerance rate at age 3 years in the United States ranges from 20% to 60% [4]. Patients with soy-induced FPIES have different tolerance rates depending on the region, with over 90% showing tolerance at 10 months in Korea [16], while the tolerance rate at age 5 in the United States ranges from 20% to 60%. In Australia, a 12.5% tolerance rate was observed at age 3 in patients with egg-induced FPIES, while in Japan, 64% of patients achieve tolerance by the age of 2. In Europe, the tolerance rate for patients with fish-induced FPIES aged between 2 and 8 years is 34% [4]. In Italy, in particular, the following average ages regarding the achievement of tolerance for the most frequently involved foods have been reported: 24 months for cow’s milk-induced FPIES [22], 30–44 months for hen’s egg-induced FPIES (respectively for cooked and raw hen’s egg) [18], and 53 months for fish-induced FPIES [19].

### 3.2. FPIES in Adulthood

In adults, the median age of FPIES onset is approximately 35 years, and there is a clear female predominance (80% of cases) [8]. The main trigger foods are shellfish (60%), bivalve mollusks, and fish (48%) [20,21].

In contrast to pediatric forms of acute FPIES, vomiting is not present in all adult patients, who may, however, present with other gastrointestinal symptoms, such as abdominal pain (present in all patients) and diarrhea (approximately 72% of cases) [1]. In addition to gastrointestinal symptoms, adults with acute FPIES may present weakness, chills, and lethargy, while hypothermia, hypotension, and dehydration are less common in the adult population than in children [8]. Adults can manifest symptoms up to 6 h after ingestion of the offending food [8].

Symptoms in adults appear to be less dramatic compared to the pediatric population, as evidenced by a study in which emergency care was required in only 7 of 25 cases [25]. Current diagnostic criteria may not be appropriate for adults, making OFC an essential diagnostic tool [9]. OFCs that have been performed in adults have followed similar protocols to those that have been published for children, and different dose schedules have been reported in adults. In the study by Crespo et al. [22] a single dose of adult serving size per day was administered on 2 non-consecutive days. However, it was noted that many adult patients declined the offered oral food challenge for the diagnosis or for assessing the achievement of tolerance because of prior severe symptoms. According to a recent systematic review, positive OFC symptoms were mostly abdominal pain (98%), followed by diarrhea (71%) and vomiting (40%). Other less-reported symptoms were lethargy (13%), nausea (9.6%), abdominal distension (9.6%), hypothermia (9.6%), weakness (7.6%), and chills (5.7%) [71].

The natural history of adult FPIES is comparatively less favorable, with tolerance developing in < 50% of cases after 1–4 years [20,23], so the treatment for FPIES is strict avoidance of the culprit food or foods. Both adult and pediatric populations with FPIES had an atopic background with symptoms of allergic rhinitis, asthma or atopic dermatitis [9,24].

### 3.3. FPIES in Adolescence

The spectrum of age of onset of symptoms due to FPIES also includes adolescence, the transitional phase of life between childhood and adulthood, from ages 10 to 19 [26].

The studies focused on adolescent FPIES and published in PubMed from 2012 to May 2025 are summarized in Table 1.

FPIES observed in adolescents and young adults may be a continuation of childhood-onset disease and usually retains the clinical features typical of the pediatric form [25]. Few data are available in the literature on FPIES in adolescence [9]. The prevalence of acute FPIES in the 14–17 age group is estimated at 0.37%, but more large epidemiologic studies are needed to describe the global impact of this condition [85]. A prospective follow-up study [25] noted that symptoms persisted in 14 of 25 patients older than 14 years of age; some of these patients reported a longstanding history of food-induced adverse reactions. In 2014, Caubet and colleagues [73] showed that FPIES was still persistent in 30–40% of children at 6 years of age.

The research on possible risk factors for persistent FPIES revealed that multiple sensitizations to foods (≥3) can cause refusal of food in patients with FPIES [27], as observed in IgE-mediated forms of food allergy [86]. Furthermore, some trigger foods, such as cow’s milk and banana, can be related to poor nutritional status [27], a condition requiring prompt nutritional counseling [28]. The market basket analysis, a data-mining technique, can help to identify associations of food triggers of FPIES and to personalize dietary recommendations [74]. The growing evidence of heterogeneity of age onset of symptoms encourages the search for diagnostic tools for early detection of FPIES. Recently, the Naples Pediatric Food Allergy (NAPFA) score allowed a group of Italian researchers to identify children with suspected FA through anamnestic and clinical features [79].

Sometimes, however, acute FPIES can begin directly in adolescence. In these cases, FPIES may present clinically with the characteristics of the pediatric form or begin as in the adult form. One of the possible causes of the onset of FPIES in teenage years could be the ingestion of foods not usually consumed in childhood, such as crustaceans, mollusks, and fish [25,30]. Therefore, in the absence of prior exposure to specific foods, the dietary changes typically occurring during adolescence may trigger the onset of symptoms compatible with FPIES [87].

A growing literature has investigated the role of several potential biomarkers of FPIES in the pediatric population to facilitate differential diagnosis with food allergies [29]. These potential biomarkers include single nucleotide polymorphism and genes of susceptibility [31], fecal eosinophilic derived neurotoxin (f-EDN) and fecal calprotectin (as indicators of gut inflammation) [32,33,34,35,36], fecal secretory IgA (f-sIgA) (as indicator of intestinal immunity) [33,35,37,38], inflammatory profiles such as IL-17, IL-10 and CXCL10 [38,39,40,41,42,81], serum C-reactive protein [88,89], procalcitonin [90,91], urinary prostaglandin metabolites [92], and hematologic indices (hematocrit, hemoglobin, platelets, and leukocytes) [80]. Another potential diagnostic contribution can be derived from the analysis of intestinal microbiota [43,44,45].

#### 3.3.1. Genetic Biomarkers

The relationship between genomic susceptibility and FPIES has been recently explored in a prospective multicenter study that analyzed data collected from 38 patients with an age range of 1–12 years and a diagnosis of acute FPIES confirmed using OFC with fish, cow’s milk, and egg as culprit food in 55%, 18.4%, and 18.4% of subjects, respectively [31]. The gene-based association test analysis identified four FPIES-related genes: “Diacylglycerol Kinase Zeta” (DGKZ), “Signal Regulatory Protein Alpha” (SIRPA), RBM8A, and ATG16L1. The transcriptome-wide association study (TWAS) analysis showed four expression quantitative trait locus (eQTL)-regulated genes potentially related to the pathogenesis of FPIES: RBM8A in the stomach and pancreas, ATG16L1 in the transverse colon, PIAS3 in the pancreas, and RPIA in the esophagus. Interestingly, the expression levels of RBM8A and ATG16L1 were higher than expected, whereas the values of PIAS3 and RPIA were lower than expected. The identification of the DGKZ gene as related to FPIES supports the critical role of the transforming growth factor β (TFG-β) pathway in the pathogenesis of FPIES. In fact, the protein encoded by DGKZ promotes signaling of TGF-β [93], a tolerogenic cytokine involved in maintaining intestinal epithelial barrier integrity by restoring enterocyte barrier function [94] and regulating IgA production [95]. The correlation analysis of co-expression revealed a functional relationship between the RBM8A gene and the filaggrin FLG gene in the tissues of the stomach and intestine [31]. Filaggrin (FLG) gene mutations are known pathogenic factors for IgE-mediated food allergies through the dysfunction of the epithelial barrier of the skin and intestine. ATG16L1, involved in autophagy, a dysregulated process in inflammatory bowel disease IBD [96], suggests the presence of common pathogenic mechanisms between FPIES and IBD. The identification of SIRPA could reflect the innate immune dysregulation observed in the acute form of FPIES, with possible migration of inflammatory cells to the gut tissue [77].

While promising, these genetic findings are based largely on pediatric populations (Table 2) and need further validation in adolescents, a group that may show distinct immune-gene expression patterns due to hormonal and developmental shifts.

#### 3.3.2. Immunologic Biomarkers

Since the first evidence of a potential pathogenetic role of an altered immunologic response, in terms of increased values of secretory IgA after ingestion of food antigens [98], subsequent reports investigated the putative role of IgA humoral response in FPIES [33,35,37,38,99]. Adel-Patient et al. found specific serum IgA levels against cow’s milk antigens significantly lower in nine children with cow’s milk (CM)-FPIES compared with six children with an IgE-mediated CM allergy. Interestingly, these differences were not dependent on food consumption or avoidance [38]. Similarly, Wada et al. observed increased levels of f-sIgA after OFCs in patients with FPIES compared with controls [35]. Conversely, an inverse behavior was observed in the comparison of serum casein-specific IgA values between twenty-six children with milk-FPIES and subjects with other-food-FPIES/controls [37]. In the recent first case-control, longitudinal study enrolling thirty-eight French children with FPIES, followed until tolerance acquisition, the detection of both serum and f-sIgA was performed for the first time [33]. f-sIgA values were significantly higher in allergic compared with tolerant patients, leading the authors to speculate a possible role of “guardian” of the gut lumen, able to counteract food allergens in active FPIES [33].

However, its behavior in adolescents remains unknown, and puberty-related changes in mucosal immunity could influence its diagnostic performance.

Cytokines and chemokines, including IL-10, IL-17, and CXCL10, have been implicated in the inflammatory response in FPIES. These molecules indicate involvement of both the innate and adaptive immune systems, yet none are currently standardized for diagnostic use. Furthermore, adolescent-specific data on cytokine profiles in FPIES are lacking, hindering their clinical translation [33].

#### 3.3.3. Gut Inflammation Biomarkers

The recent literature is unravelling the complex immunological intertwining underlying the intestinal mucosal inflammatory state found in FPIES patients, with involvement of innate and adaptive immunity, resulting in impaired barrier function [100]. Notably, EDN is a granule-derived alarmin that reveals the severity of eosinophilic gastrointestinal inflammation [34,101]. It is a very easy-to-measure, non-invasive fecal biomarker, stable at room temperature for at least 7 days, with higher concentrations in younger children, even with cow’s milk FPIES [35,101,102]. However, f-EDN cannot be considered a marker of chronic intestinal inflammation in FPIES because this molecule increases after exposure to causative food during OFC, due to the release and recruitment into the intestinal lumen of eosinophils but normalizes shortly after the challenge [33]. To date, f-EDN has not been validated in adolescents, and normative values for this age group are lacking.

Similarly, calprotectin, a cytosolic Ca^2^+/Zn-binding protein present in neutrophils and monocytes, with antimicrobial, immunomodulatory and anti-proliferative action [103], is elevated in fecal samples from patients with FPIES, but concentrations reduce after OFC [33].

In pediatric FPIES, f-Cal levels increase acutely after oral food challenges (OFCs) and normalize after symptom resolution, reflecting transient gut inflammation rather than ongoing pathology [33]. However, f-Cal remains unvalidated among adolescents, and its utility may be confounded by pubertal immune changes.

#### 3.3.4. Microbiome-Related Biomarkers

The intestinal microbiota has emerged as a key player in FPIES pathophysiology. Ongoing research aimed at better understanding the pathophysiology of FPIES has led to exploring the intestinal microbiota [43,44,45]. Relevant was the finding of reduced diversity of the fecal microbiome in infants affected by FPIES compared with controls, with reduced representation of commensal species of *Bifidobacterium* and *Clostridium* [97].

A recent study analyzing fecal samples from 17 children with FPIES (mean age ~7.5 years) revealed significant microbial alterations compared with healthy controls: reduced diversity, decreased abundance of *Lactobacillaceae*, *Leuconostocaceae*, and *Ruminococcaceae*, and increased levels of *Lachnospiraceae* [45]. These shifts suggest a pro-inflammatory microbiota configuration with diminished production of short-chain fatty acids (SCFAs), crucial for gut immune tolerance. Previously, the use of antibiotics during pregnancy (but not during breastfeeding or before conception) was found to be significantly associated with an increased risk of FPIES in newborns [104]. This suggests that alterations in the prenatal maternal microbiome may influence neonatal colonization and the child’s immune system development, increasing the risk of developing FPIES.

No studies to date have examined microbiota in adolescents with FPIES, making it unclear whether these dysbiotic patterns persist, evolve, or resolve during puberty.

Validation studies of these biomarkers in the adult population and even more so in adolescents are increasing [77,99,105,106]. This complicates the understanding of the pathophysiological mechanisms of FPIES in these populations.

The Naples Pediatric Food Allergy (NAPFA) score integrates clinical history and symptom patterns to support food allergy diagnosis and has demonstrated strong predictive accuracy in pediatric populations up to 12 years of age [79]. Given that adolescence begins at age 10 according to the World Health Organization, this tool may hold future utility in the early adolescent population (ages 10–12). However, it has not yet been validated in older adolescents (13–19 years), underscoring the need for age-stratified studies to assess its diagnostic performance across the full adolescent spectrum.

Validating FPIES biomarkers in adolescents faces multiple hurdles:

- Small sample sizes, due to the rarity of FPIES in this age group, limit statistical power.
- Puberty-related immune modulation complicates the interpretation of mucosal and systemic immune markers.
- Phenotypic heterogeneity: adolescent FPIES can resemble pediatric or adult-onset forms, increasing variability.
- Lack of age-specific normative data impairs the diagnostic precision of existing biomarkers.
- Absence of longitudinal, age-stratified studies leaves key developmental transitions unexplored.

While several biomarkers and genetic signatures (Table 2) show promise in pediatric FPIES, their applicability to adolescents remains speculative due to limited data. Adolescents represent a unique physiological window, with distinct immunological and microbial characteristics. Future research should focus on age-specific validation of gut inflammation markers, immunologic mediators, and microbiota-derived patterns to enhance diagnostic precision and improve management strategies for adolescent FPIES.

For patients who continue to manifest acute FPIES reactions once the average age for acquiring tolerance for any given food has passed, the clinical evolution is not well known. The evaluation of (1) clinical latency between symptom onset and diagnosis, (2) multiple sensitizations to foods [82], and (3) different times for the development of tolerance depending on the specific FPIES trigger food [83] can help to optimize the management and obtain clinical remission.

Although these are non-IgE-mediated forms of allergy and usually have a favorable course, for pediatric forms, protocols with increasing doses of the offending food to be administered at home from very small amounts (“ladder”) [50] have been described to promote the acquisition of tolerance. Twenty-one patients with mild FPIES were started on the Canadian Egg Ladder, and 90.5% of patients completed the ladder, tolerating a serving size of cooked egg, over a median duration of 7 months. Likewise, oral desensitization with culprit food could be a therapeutic strategy for those patients with persistent forms of FPIES and who have exceeded the average age of acquiring tolerance, as proposed and successfully conducted in one case [46]. No cases of oral desensitization have been described in adult or adolescent forms of FPIES where strict avoidance remains the only therapeutic option.

The diagnostic criteria established for the pediatric population may be applied to adolescent patients presenting with a persistent form of FPIES or a new-onset FPIES that exhibit clinical features typical of the pediatric phenotype. In contrast, if the clinical presentation is consistent with the adult form, these criteria are not applicable.

OFC remains a cornerstone for the diagnosis of acute FPIES or for assessing the achievement of tolerance. Recent evidence highlights the importance of the setting of OFC and the risks related to performing the test at home or in the outpatient clinic [47].

The OFCs performed in adolescents usually follow the same protocol used for pediatric patients, as no age-specific protocols have been established. To date, there is no evidence in the literature indicating whether there is an increased risk of serious reactions in adolescents.

In adolescent FPIES with the characteristics of the adult form, symptoms appear to be less severe compared to the pediatric population, and the clinical presentation requires differential diagnosis with other conditions. Therefore, the use of age-specific diagnostic algorithms may support more accurate identification of FPIES [48,49]. Interestingly, an atypical form of adolescent FPIES associated with a congenital glycosylation disorder was described for the first time in Australia [107].

Table 3 summarizes the characteristics of the different clinical presentations according to age. The characteristics reported in the table for adolescents are based on data described in the article by Gonzalez-Delgado et al. [25].

**Figure 2:**
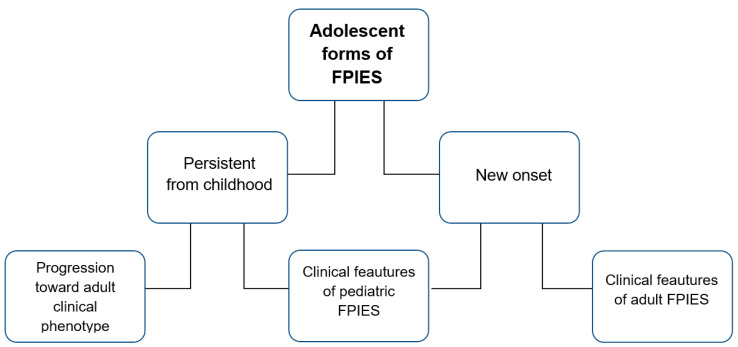
The possible presentations of acute FPIES in adolescents and young adults.

Figure 2 shows the possible presentations of acute FPIES in teenagers and young adults.

## 4. Future Directions

Additional studies are needed to clarify the spectrum of clinical presentation of adolescent FPIES and its evolution over time. The latency between symptomatic onset and diagnosis can be significant [108]. To date, the best way to remove the veil of Maya that hinders the understanding of the natural history of FPIES from childhood, through adolescence to adulthood, is undoubtedly the creation of international registries that, with a multi-specialist approach, systematically collect data and follow over time patients affected by FPIES [109]. This approach could improve therapeutic options such as oral desensitization aimed at achieving tolerance.

For adolescents diagnosed with chronic conditions, the transition from pediatric to adult care goes far beyond simply shifting medical oversight. It involves a gradual, well-structured process that focuses on improving their understanding of the disease, building their confidence in managing it, and encouraging greater independence in health-related decisions. Although there is currently limited research dedicated specifically to transition strategies for FPIES, early reports suggest that these patients face distinct challenges during this critical period, especially related to dietary restrictions, the need for coordinated allergy care, and ongoing psychological support [110].

Given the lack of transition protocols designed specifically for FPIES, it may be helpful to adapt successful strategies from other chronic gastrointestinal conditions, particularly IBD. Experience with IBD transition programs underscores the importance of early patient involvement, gradual assumption of responsibility, and close collaboration between pediatric and adult medical teams to ensure continuity of care [111]. Transition pathways should therefore take into account not only patients’ clinical stability but also their emotional readiness, psychological well-being, and social support network [112,113].

A practical adaptation of IBD transition models to FPIES would involve the implementation of age-specific education modules, starting from early adolescence, focusing on understanding of the disease, food avoidance strategies, and communication with healthcare providers. A multidisciplinary transition team, including pediatric and adult gastroenterologists, allergists, dietitians, psychologists, and a transition coordinator, should support this process throughout. The transition should be structured in three flexible phases: preparation (assessment and goal setting), engagement (shared consultations and increasing autonomy), and transfer (independent adult care, supported by digital tools) [111,114].

Adolescents with chronic illnesses, including IBD, are known to have low adherence rates, often exceeding 50%, due to risk-taking behavior, social pressures, and reduced parental supervision [115]. These challenges are relevant for PFIES as well, where strict dietary adherence is critical. Moreover, anxiety and reduced quality of life among adolescents with food allergies further justify the need for tailored psychological support within transition models.

Encouraging resilience and promoting self-efficacy are also essential elements in supporting adolescents as they take on a more active role in managing their health [116,117].

Consequently, it would be beneficial for healthcare teams managing FPIES patients to design personalized, multidisciplinary transition programs. These programs should ideally involve pediatricians, allergists, gastroenterologists, nutritionists, mental health professionals, and patient advocacy groups.

Transition is a critical time because age changes, needs change, and the options available change. Therefore, communication cannot be seen as a simple exchange of information, but rather a structured process, organized in dedicated settings. It must have appropriate and shared content (the care plan must be based on the recommendations of common guidelines) and formalized by providing a care plan detailing what has been received, what could happen, and what can be done [118]. Providing adequate information and a follow-up program is one of the methods needed to reduce anxiety and maintain adherence to checkups while avoiding over-medicalization.

Finally, interoperable digital models based also on telemedicine, artificial intelligence, and E-Health tools could improve the clinical management of food allergies during this delicate phase of life. Such comprehensive and collaborative approaches may significantly improve health outcomes, treatment adherence, and quality of life for young people as they move toward adult care.

## 5. Conclusions

Acute FPIES in adolescence is not a well-known form, and few data is available in the literature. It is important to be able to diagnose it at its onset because it may not present the classic features of pediatric forms but those of adults. Currently, the diagnostic criteria of FPIES may not recognize all these patients.

## Figures and Tables

**Table 1:** List of publications on adolescent FPIES over the past fourteen years.

Adolescent FPIES	Study Country Reference no.°	Type of Study	Age Range [Years]	No. of Participants Enrolled	Relevant Findings
Clinical Features	Spain [25]	Prospective follow-up study	≥14	25	Clinical features of FPIES related to seafood
	Spain [30]	Retrospective study	4–12	80	Clinical features of FPIES related to fish
	US [72]	Retrospective chart review	0–18	210	Comparative study of 2 FPIES cohorts diagnosed during different guidelines and recommendations
	US [73]	Ambispective study	0.5–45	160	Clinical features in a large cohort of patients
	US [74]	Retrospective analysis of electronic medical records	0–17	210	Identification of patterns and associations in FPIES through Market Basket Analysis
	Italy [75]	Case report	14	1	The first case of mollusk (oyster) FPIES in an adolescent
	Spain [76]	Retrospective Study	0.11–14	16	Clinical and developmental characteristics of a Spanish case series
Nutritional status	Italy [28]	Non-randomized, prospective intervention study	0–14	100	Deleterious impact on nutritional status and utility of dietary counseling
	US [27]	Retrospective study	All	203	Multiple sensitizations to food as risk factors for food aversion. Persistent FPIES in twenty-one percent of patients between 6 and 17 years of age
Pathophysiology	US [77]	Cross-sectional study	1–21	30	Systemic innate activation and redistribution of lymphocytes related to reactions to foods
	US, Spain and Poland [78]	Cross-sectional study	2–16	20	Adenosine and serotonin pathways in gastrointestinal inflammation
Diagnosis	Italy [79]	Cross-sectional study	0–14	627	Multivariable regression model to predict suspected FA
Genetic Biomarkers	Spain and Italy [31]	Multicenter retrospective study	1–12	38	Identification of single-nucleotide polymorphisms and genes capable of revealing susceptibility to FPIES
Biomarkers	Japan [32]	Observational study	0–15	27	Potential use of fecal hemoglobin, fecal lactoferrin and fecal calprotectin to assess intestinal inflammatory status
	Spain and Italy [80]	Observational multicenter prospective study	0–18	81	Potential diagnostic role of hematocrit, hemoglobin, platelets, and leukocytes
	US and Poland [81]	Cross-sectional study	1.5–16	11	Acute reactions associated with IL-17 inflammatory signature
OFC safety	Italy [47]	Retrospective study	0–17	202	OFC is not safe enough for acute FPIES at home
Tolerance	Italy [82]	Multicenter retrospective comparative cohort study	0–13	123 total (21 FPIES)	Identification of non-modifiable and modifiable factors influencing the time of immune tolerance acquisition and the occurrence of allergic march.
	Sweden [83]	Prospective follow-up study	0–16.5	113	Achievement of tolerance
Therapy	Italy and Australia [84]	Retrospective case series	0.4–14	66	Potential use of ondansentron

Abbreviations: FPIES, Food protein-induced enterocolitis syndrome; FA, food allergy; OFC, Oral Food Challenge; US, United States; N/A, not applicable.

**Table 2:** List of genes, SNPs, and gut microbiota as biomarkers for FPIES.

Type	Gene/SNP/Microbiota	Function/Role	Reference
Gene	DGKZ	Promotes TGF-β signaling by regulating epithelial barrier function and IgA production	[31,93,94,95]
Gene	SIRPA	Immunoinhibitory receptor, associated with innate immune dysfunction, upregulated in acute FPIES reactions	[31,77]
Gene	FLG	Functions in the epithelial barrier of skin and intestine; a crucial risk factor for food allergy	[31]
Gene	↑ATG16L1	Involved in autophagy, associated with inflammatory bowel disease (IBD). Higher expression levels in the transverse colon are associated with FPIES	[31,96]
Gene	↑RBM8A	Involved in food allergies and thrombocytopenia with absent radius (TAR) syndrome. Higher expression levels in stomach and pancreas are associated with FPIES	[31]
Gene	↓PIAS3	Regulates immune response; potentially associated with FPIES pathogenesis in the pancreas	[31]
Gene	↓RPIA	Regulates cellular metabolism via the pentose phosphate pathway; potentially associated with FPIES in the esophageal tract	[31]
Microbiota	↓Bifidobacterium, ↓Clostridium	Modulates intestinal immune response and contributes to epithelial barrier regulation	[97]
	↓short-chain fatty acids (SCFAs)	Gut immune tolerance	[45]

↑: increase of expression; ↓: decrease of expression.

**Table 3:** Characteristics of FPIES presentations in pediatric, adult, and adolescent patients.

	Childhood	Adulthood	Adolescence
Predominance	Slight male or equal sex distribution	Female	Female
Main trigger foods	Cow’s milk, fish, egg, rice, soy	Shellfish, fish	Shellfish, fish
Prevalent symptoms (in order of frequency)	Vomiting (100%) pallor, hypotonia, lethargy	Abdominal pain (100%) diarrhea, vomiting	Abdominal pain, diarrhea
Diagnosis	Diagnostic criteria panels	Clinical history and OFC	Clinical history and OFC
Natural history	Favorable	Less favorable	Less favorable
